# A scoping review of initiatives to reduce inappropriate or non-beneficial hospital admissions and bed days in people nearing the end of their life: much innovation, but limited supporting evidence

**DOI:** 10.1186/s12904-020-0526-2

**Published:** 2020-02-27

**Authors:** Ros Taylor, Jonathan Ellis, Wei Gao, Liz Searle, Kate Heaps, Robert Davies, Claire Hawksworth, Angela Garcia-Perez, Giles Colclough, Steven Walker, Bee Wee

**Affiliations:** 1 Hospice UK, London, UK; 2 Cicely Saunders Institute, London, UK; 3 Keech Hospice Care, Luton, UK; 4 Greenwich & Bexley Hospice, London, UK; 5 Stgilesmedical Ltd, The Vestry House, St Giles High Street, London, WC2H 8LG UK; 6 Stgilesmedical GmbH, Berlin, Germany; 7 McKinsey & Company, London, UK; 8 Harris Manchester College, University of Oxford, Oxford, UK; 9 Sir Michael Sobell House, Oxford University Hospitals NHS Foundation Trust, Oxford, UK

**Keywords:** Palliative care, Home care, Hospital care, Nursing home care, Service evaluation, Supportive care

## Abstract

**Background:**

Hospitalisation during the last weeks of life when there is no medical need or desire to be there is distressing and expensive. This study sought palliative care initiatives which may avoid or shorten hospital stay at the end of life and analysed their success in terms reducing bed days.

**Methods:**

Part 1 included a search of literature in PubMed and Google Scholar between 2013 and 2018, an examination of governmental and organisational publications plus discussions with external and co-author experts regarding other sources. This initial sweep sought to identify and categorise relevant palliative care initiatives. In Part 2, we looked for publications providing data on hospital admissions and bed days for each category.

**Results:**

A total of 1252 abstracts were reviewed, resulting in ten broad classes being identified. Further screening revealed 50 relevant publications describing a range of multi-component initiatives. Studies were generally small and retrospective. Most researchers claim their service delivered benefits. In descending frequency, benefits identified were support in the community, integrated care, out-of-hours telephone advice, care home education and telemedicine. Nurses and hospices were central to many initiatives. Barriers and factors underpinning success were rarely addressed.

**Conclusions:**

A wide range of initiatives have been introduced to improve end-of-life experiences. Formal evidence supporting their effectiveness in reducing inappropriate/non-beneficial hospital bed days was generally limited or absent.

**Trial registration:**

**N/A**

## Background

For patients, being in a hospital bed during the last weeks of life when they have no medical need or desire to be there is distressing and expensive [1–3]. Patients with incurable conditions are often admitted with little or no benefit to their clinical state [4]. While there may be mitigating circumstances, e.g. providing respite care, such inappropriate or non-beneficial use of resources may be regarded as a failure of the system [4–6]. This problem is likely to intensify as the number of people dying increases along with their age at death [7]. Also, people are living longer with multiple morbidities, necessitating additional resources [8]. Several reports have sought to address inadequacies in care provision [9–13].

Place of death is important for some patients and their families. Whereas the literature suggests many people want to die at home, this preference may vary with age, disease state and care needs [14, 15]. For many patients, their eventual place of death may not match individual preferences [16]. An international comparison found that more than 50% of deaths occur in hospital, ranging from 78% in Japan to 20% in China [17].

Dying in hospital is likely to be the costliest component of care in the final months of life [18]. Data from England collected between April 2009 and March 2012 showed that, on average, adults in the last year of life experienced 2.3 hospital admissions accounting for 30 bed days, with 57% of usage occurring in the last 3 months of life [19]. The costs of UK hospital admissions in 2011 for adults in the last year of life was estimated to have been £1.3 billion, with a report from the Netherlands suggesting that this cohort was responsible for 10–12% of total health expenditure [6, 20].

Such data has caused some to speculate that the economic burden could be minimised by introducing initiatives designed to avoid or shorten unnecessary admissions at the end of life [6, 21]. An example of what could be achieved comes from a retrospective analysis of 483 patients who died within 1 year of admission to two UK hospitals. This analysis concluded that 35 admissions were potentially avoidable and could have saved the hospitals £5.9 million per year [5]. Had hospital stays been shortened across all 483 patients by 14%, the savings would have been substantially greater, at an estimated £47.5 million. Others consider these figures to be an underestimate, suggesting that 20–40% admissions in the last year of life could be avoided [22, 23]. It is acknowledged that shifting the burden of care would result in increased family and community care costs [24].

One problem is the lack of accurate data to compare the costs of delivering palliative care across different settings [6, 18]. In the United States, Medicare and Medicaid data show that costs vary widely but there are advantages in the form of actual reimbursement by the insurer [18]. In comparison, the widely quoted UK target price for one specialist palliative care National Health Service (NHS) bed day of around £412 represents the national tariff paid by a commissioner, rather than the actual cost to the healthcare provider [18, 25]. Estimates for community and hospice costs are generally considered even less reliable. A more useful proxy measure for the success of a palliative care innovation, may be a reduction in hospital bed days when hospital admissions are not medically indicated or in accordance with patients’ or families wishes.

There are many reports of palliative care initiatives worldwide that could have an impact on hospital admissions and bed days. These initiatives often involve the hospice movement, which has shown itself proactive in developing new services. Feedback from specialists suggests that the range of services that could benefit patients and their families is not widely appreciated outside of senior palliative care circles. Similarly, limited information is available regarding the effectiveness of many initiatives. As discussed, measuring resource usage may be preferable to estimating costs as an outcome measure. Consequently, the two main aims of this research were to identify initiatives which may avoid or shorten unnecessary or unwanted hospital admissions at the end of life and analyse their success in terms of a reduction in bed days. Data from this study will inform the ongoing HOspice-Led Innovations Study To Improve Care (HOLISTIC) project [26].

## Methods

This study comprised two parts. The first part sought to identify and categorise initiates which may influence hospital admissions and bed days. The second part explored objective reasons for success of the intervention by reducing bed days and how initiatives might be upscaled or transferred elsewhere. Both parts involved a multiple author search of articles in English in PubMed and Google Scholar between 1st January 2013 and 31st November 2018, an examination of governmental and organisational publications, a hand-search of documents looking for additional information, and discussions with external and co-author experts regarding other sources of information.

### Part 1

We sought to identify and classify established initiatives which were considered likely to have a quantifiable impact on hospital admissions and bed days at the end of life. Search terms included ‘avoidable/inappropriate hospital admissions’, ‘cost/economics at the end of life’ or ‘hospital discharge’. Our initial search resulted in the identification of ten initiatives grouped under three broad headings.

### Part 2

A more detailed literature search was conducted separately for each initiative identified in Part 1, using the search terms ‘end-of-life’, ‘palliative care’, ‘hospital bed days’, ‘hospice’, ‘nursing home’, ‘reduction’, ‘decrease’, ‘innovation’, ‘hospital admission’, and ‘length of hospital stay’. The contents of these publications were hand searched for by other relevant data sources. Only those presenting data on hospital utilisation and/or duration of stay were included. Hospital utilisation was defined as admission to either hospital and/or emergency departments, whatever the cause, in patients considered to be in the last year of life. Further exclusions were made if the study language was non-English and/or a paediatric population was included. We also removed any studies where the palliative intervention was considered routine practice. After the selected publications were examined, the methodology was refined to seek information on the steps required for initiatives’ introduction, sustainability and upscaling.

## Results

### Part 1

**Fig. 1:**
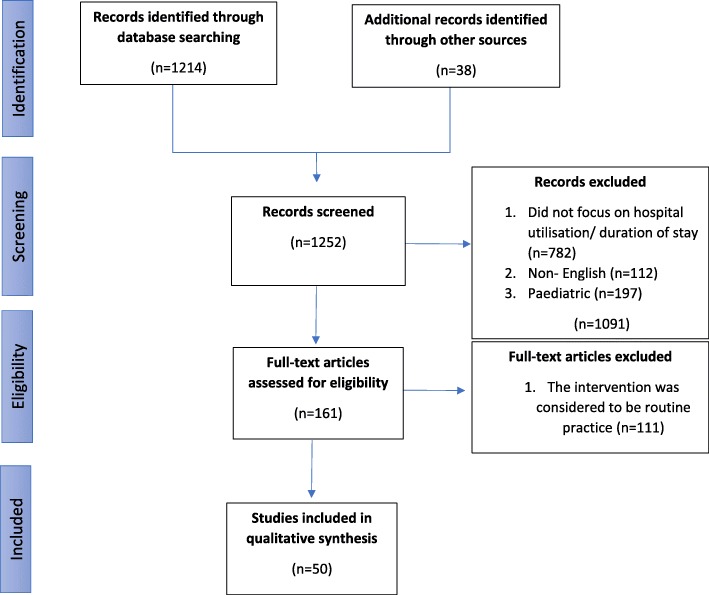
PRISMA diagram

A total of 1252 abstracts were reviewed by one author, resulting in the identification of ten categories of initiatives grouped under three broad headings (Fig. 1, Table 1): 1;‘Facilitating entry into the hospice and community care system’ [27], 2a-2 g; ‘Preventing admission’ [28–74] and 3a&3b; ‘Facilitating discharge’ (Table 1) [56, 66, 72, 75–78]. Classification was reviewed by a second author. Because of variations in terminology used to describe similar activities and the multifactorial nature of some initiatives, a degree of judgement was required when determining which group the initiative should be assigned to. Differences of opinion were resolved by discussion among the authors.

**Table 1:** Classification of innovations which may avoid inappropriate or non-beneficial hospital admission and/or reduce bed days for patients at end of life: definitions and sources of information

Classification of innovation		Types of innovation and examples of what is involved		Publications providing quantitative data included in this review
1	Facilitating entry into the hospice and community care system	Single point of access	Access to a range of medical, nursing and social care services for patients, carers and healthcare professionals via a single telephone number.	[27]
2	Preventing hospital admission	2a; Care home innovations	A mixed group, including training programmes for nursing home staff.	[28–37]
		2b; Palliative care support in the community, 24/7 hospice at home service	Coordinated palliative care delivered in the patient’s home through regular visits by specialist medical and nursing staff, often in association with a hospice.	[38–55]
		2c; 24/7 helpline	Support for patients, carers, paramedics and non-specialist doctors and nurses.	[27, 39, 46, 48, 56]
		2d; Telehealth/ telecaring	Provision of healthcare remotely using telecommunication technology.	[36, 37, 39, 57, 58]
		2e; Ambulance staff education	Training for better communication and decision-making when attending patients at the end of life.	[59]
		2f; Integrated palliative care	Coordinated input from different healthcare professionals and caregivers.	[60–73]
		2 g; Palliative care outreach in rural areas	Palliative consultant attends the patient in their own home and coordinates care.	[56, 74]
3	Facilitate discharge	3a; Hospital or emergency department-based discharge service	Patients requesting or considered suitable for discharge into supported care (community, nursing home, hospice) are identified by a hospital-based doctor or nurse who facilitates onward management.	[56, 66, 72, 75–77]
		3b; Nurse-led palliative care inpatient capacity expansion	Offering additional nurse-led beds for less complex patients.	[78]

### Part 2

#### Overview

Fifty publications provided data on hospital utilisation and/or hospital stay. Selected publications often described initiatives with multiple components, resulting in them sometimes being included in more than one category. Studies were generally small and retrospective in nature. Most researchers claimed their services delivered benefits—in descending frequency order, these benefits were, palliative care support in the community (including hospice at home services), provision of integrated care, out-of-hours telephone advice, care home education and telemedicine. Nurses and hospices were central to many initiatives. Elements for success, sustainability, upscaling or adoption elsewhere were not generally addressed in the publications.

#### 1. Single point of access

Several organisations offer Single Point of Access (SPA) services for people at the end of their life, but published evidence of the services’ benefits is limited. The Sue Ryder organisation published an evaluation report of a pilot initiative, ‘Partnership for Excellence in Palliative Support’ (PEPS) [27]. PEPS is a 24-h telephone SPA service bringing together 15 organisations across East England, UK, with senior nurses as the first point of access.

During the pilot period, 1051 patients were registered with Partnership for Excellence in Palliative Support. The majority (65%) who died, were supported to die at home, with only 11% dying in an acute hospital. Information from a sample of patients was compared with hospital activity data sets before and after registration and suggested that the introduction of a Single Point of Access service could result in 30% fewer admissions, a 30% shorter stay and cost reductions of around £300 per admission.

#### 2a; care home innovations

Care home residents at the end of their life are sometimes transferred to hospital when another course of action might be more appropriate [1], possibly due to a knowledge gap among all grades of care home staff which could be addressed by additional training. The problem was explored in three studies using a mixed-methods approach (Table 2) [28–30] or quasi-experimental design [31] or as part of a randomised controlled trial [32]. Training varied in terms of duration, frequency and who delivered it. Common topics were, recognition of the end of life, advance care planning, optimising communication with residents and their families and documenting changes in patient preferences and condition.

**Table 2:** Care/nursing home education programmes

Author, date and journal	Study design	Sample and setting	Research focus	Relevant results	Wider implications	Limitations
Garden et al.; 2016; *Clinical Medicine* [28]	Mixed methods.	7 dementia care homes in Boston, UK, registered for the intervention programme, commencing in March 2011. 250 staff were trained.	To explore the impact of a care home educational programme (Bromhead Care Home Service) on hospital admissions/place of death for palliative residents.	From baseline, admissions were reduced by 66 in the first year and 111 by the third year, which represented a 55% decrease. 102 care plans were completed by April 2013. 4 of 102 patients wanted to die in hospital. Of the 68 residents who died during follow up, 67 died in preferred place.	Care home educational programmes such as the Bromhead Care Home Service could reduce hospital admissions for dementia patients.	Evaluation done in-house. Evaluation was done retrospectively, chance of recall bias.
Livingston et al.; 2013; International Psychogeriatrics [29]	Mixed-methods retrospective.	120 Jewish residents with dementia and 90 nursing and care staff at a care home in London, UK. The educational intervention was a 10-session, manualised, interactive staff training programme.	To analyse the impact of a care home educational programme on outcomes, including place of death.	Significant increase in residents dying in the care home vs. hospital (47% vs.76%, *p* = 0.02). Trend toward a decrease in the number of days spent in hospital during the last 3 months of life after intervention (median 4 vs. 1.25).	Increased care home education is associated with a decrease in hospital deaths and hospital days during the last months of life.	May not replicate in non-Jewish care homes. Non-randomised.
Rantz et al.; 2015; Journal of Nursing Care Quality [30]	Mixed-methods longitudinal.	16 American nursing homes where hospital rates were among the country’s highest for hospital 30-day readmission and the Missouri Quality Initiative^a^ intervention was in place.	To examine the Missouri Quality Initiative’s effect on avoidable hospital admissions (other variables measured were polypharmacy and antipsychotic medication use, care discussions and completion of Advance Directive and the introduction of secure communication for electronic transfer of health information to other services).	Transfer rates steadily declined approximately 9 months after programme implementation. Hospital transfers per 1000 days were 1.7 in 2014 and 1.3 in 2015 and was on track for 1.1 in 2016. Education was a theme that care staff believed reduced hospital admissions. They believed the advanced practice registered nurses were key in improving assessment skills through training. All staff members were willing to be involved in the programme, aiding the reduction further. Reports provided a visual picture of nursing staff progress.	The Missouri Quality Initiative reduced unnecessary hospital admissions in a nursing home setting.	
Chapman et al.; 2016; *BMJ Supportive & Palliative Care* [31]	Quasi-experimental, quantitative comparative.	250 Australian palliative care patients spread across 4 residential facilities over a 6-month period. 77 were included in the intervention group. The control group of 173 decedents was retrospective.	To examine the effect of a ‘Palliative Care Needs Rounds’ programme (supporting clinical decision-making, education and training) on hospital utilisation.	The intervention group had on average a shorter length of hospital stay, than those not enrolled in the programme (1.9 vs. 4.8 *p* = 0.02). No significant difference in number of hospital admissions.	Care home education could decrease the length of hospital stay.	Historical data used for the control group. Some residents were excluded due to incomplete data.
Kane et al.; 2017; *JAMA Internal Medicine* [32]	Clustered randomised clinical trial.	36,717 residents at 85 nursing homes, 33 intervention sites and 52 control sites that had not previously implemented the INTERACT (Interventions to Reduce Acute Care Transfers) quality improvement programme.	To establish whether training and support for implementation of a nursing home quality improvement programme reduce hospitalisations and ED visits.	There was no significant difference between intervention and control nursing homes regarding overall hospital admissions, 30-day readmission rates or emergency department visits that resulted in hospitalisation. Intervention homes showed a 6.5% reduction in overall hospitalisations, and 15% reduction in potentially avoidable hospitalisations compared with the pre-intervention rate amongst intervention homes.	Training and support for INTERACT implementation had no impact on hospitalisation or emergency department visits.	The original sample contained a substantial number of nursing homes that reported prior use of INTERACT.

^a^*The MOQI is 4-year intervention which assists care-staff training using advanced practice registered nurses, care transitions, health information technology and assessment tools*

Staff education was generally linked to a reduction in hospital admissions or a shorter length of hospital stay. Garden et al., for example, reported a 55% decrease in admissions compared to baseline, 3 years after implementation [28]. Livingston et al., found that the average number of days spent in hospital in the last 3 months of life decreased from four to 1.25 days [29]. Chapman et al., reported a 3.22-day decrease in length of stay, equating to a 67% reduction in bed days for residents involved in the intervention, but having no effect on the number of admissions [31]. Other demonstrable benefits, were an increased number of deaths in the care home compared with the hospital [29], and care home staff feeling ‘uplifted’ and ‘empowered’ as a result of the intervention [30].

These favourable results are at odds with the findings of a randomised US study designed to evaluate the effects of the high-profile ‘Interventions to Reduce Acute Care Transfers’ (INTERACT) quality improvement programme [33]. While there was a trend in favour of additional staff training aimed at improving care at the end of life compared with the pre-intervention period, Kane et al., found no significant difference in admission rates between nursing homes randomised to receive INTERACT training and a control group [32].

Other initiatives have been introduced to support care home residents, such as the ‘Enhanced Health in Care Homes Vanguard’ programme in the United Kingdom. While generally considered beneficial, these initiatives’ effect on hospital admissions has been variable. One simple initiative, the introduction of ‘red bags’ containing all relevant health-related paperwork and medication that travel with the patient, helped simplify hospital transfers and reduced length of stay for residents in Sutton from a local average of 14 days to 9.2 days [34]. Meanwhile, the introduction of multidisciplinary teams in Newcastle and Gateshead saw an increase in non-elective admissions, possibly related to increased symptom recognition [35]. A Vanguard project in Airedale that implemented telemedicine consultations for care home residents showed a 15% decrease in emergency admissions for homes that infrequently used the service, compared with a 10% increase for homes classified as high usage [36]. Also, a prospective observational study in Massachusetts care homes demonstrated that a group receiving telemedicine support, saw greater cost savings and hospitalisation reduction than the control group, especially where there was active engagement with the project [37].

#### 2b; palliative care support in the community

We identified 18 studies that included a focus on the impact of palliative care support in the community (e.g., hospice at home services) on hospital utilisation (Table 3). As this is a heterogeneous group of initiatives which may include hands-on nursing care, specialist advice from multidisciplinary teams and emergency intervention, we have not sought to separate them. Ten studies were retrospective [39–41, 44, 47–51, 53], five were prospective cohort studies [38, 42, 52, 54, 55] and three were described as population studies [43, 45, 46].

**Table 3:** Hospice at home

Author, date and journal	Study design	Sample and setting	Research focus	Relevant results	Wider implications	Limitations
Wong et al.; 2013; Annals, Academy of Medicine, Singapore [38]	Quantitative, prospective cohort study.	44 Singaporean patients with advanced heart failure enrolled in an Advanced Care Programme between July 2008 and July 2010.	To evaluate the impact of a home-based Advanced Care Programme on healthcare utilisation in the last stages of life for advanced heart-failure patients.	Mean all-cause and heart failure related admissions prior to Advanced Care Programme enrolment were 3.6 and 2.0 episodes. Following admission to the programme, these dropped to 1.0 and 0.6 (*p* < 0.0001), representing 72 and 70% reductions, respectively. After follow-up duration, adjusted hospital admission episodes were 1.2 and 0.5 (*p* < 0.0001).	Home-based palliative care has shown reduced hospital utilisation for patients with heart failure.	Small sample size. No control group. Did not account for placebo effects of a palliative programme.
Lustbader et al.; 2017; Journal of Palliative Medicine [39]	Quantitative, retrospective, comparative analysis.	82 US Medicare Shared Savings Program accountable care organisation deceased patients compared with 596 patients receiving usual care between October 2014 and March 2016.	To evaluate the impact of a home-based palliative care programme within an accountable care organisation on cost and resource utilisation during the final 3 months of life.	A 34% reduction in hospital admissions during the final month of life was observed in hospital-based palliative care-enrolled patients. Hospital-based palliative care was also linked with a reduction in emergency department visits per 1000 patients compared with standard care (878 vs. 1097).	Hospital-based palliative care in an accountable care organisation is linked with significant cost savings and fewer hospital admissions.	Lack of minority populations. No case-matched control group (although disease burden was quantified and equal for both groups, *p* = 0.4).
Spilsbury et al.; 2017; PLOS ONE [40]	Quantitative, retrospective cohort study.	12,763 Western Australian patients who died from cancer, and 7 from non-cancer conditions.	To investigate how community-based palliative care is associated with a reduction in acute care health service use.	During the intervention period, hospital admissions dropped by 34% and the mean length of stay decreased by 6%. For patients < 70 years, receiving community-based palliative care was linked with a reduced rate of hospital admissions approximately 5 months before death, and in those > 70 years, the reduction was observed up to a year before death.	Hospital admission rates were reduced when patients were receiving community-based palliative care. These effects could be seen within 5 months of death and earlier in older patients.	Lack of detail on intervention intensity. Palliative care may have been different for those living in rural and remote areas.
Seow et al.; 2014; British Medical Journal [41]	Quantitative, pooled analysis of a retrospective cohort study.	6218 Canadian cancer patients, of whom 3109 received community-based palliative care administered by one of 11 specialist palliative care teams (intervention)^a^, and 3109 received standard community-based palliative care during the last 2 weeks of life (control).	To understand the pooled effect of exposure to any one of 11 palliative care teams operating at patients’ homes.	970 patients from the intervention group visited a hospital, compared with 1219 from the control group (31.2% vs. 39.3%, p < 0.001). 896 patients from the intervention group were admitted to the emergency department, compared with 1070 in the control group (28.9% vs. 34.5% *p* < 0.001). The pooled relative risks of being admitted to hospital and an emergency department in late life for the intervention versus control group are 0.68 and 0.77, respectively. Fewer intervention patients died in hospital than control patients (503 vs. 887, p < 0.001).	Palliative care teams appear to reduce both hospital and emergency department admission, as well as the amount of hospital deaths.	Propensity scores cannot account for unmeasured variables such as preferred place of death or existing care. Generally, cancer patients only.
Seow et al.; 2016; Journal of Pain and Symptom Management [42]	Quantitative, retrospective cohort study.	54,576 Canadian decedents who used home care nursing services during the last 6 months of life.	To explore the link between the home care nursing rate and emergency department visit rate in the following week during the last 6 months of life.	Patients who received the intervention during any week had a 31% lower emergency department visit rate during the following week than did patients receiving standard care. During the last month of life, those receiving home care end-of-life nursing and standard care for more than 5 h per week were associated with a decreased emergency department visit rate of 41 and 32%, respectively, compared with patients with 1 h of standard nursing per week.	There is a temporal association between receiving home-based end-of-life nursing care in a specific week during the last 6 months of life and a reduced emergency department visit rate. Higher levels of both home-based end-of-life care and standard care are also linked with a lower emergency department visit rate.	Study does not imply causality. The database does not hold other important factors which may influence emergency department visit rates.
Gagnon et al.; 2015; Journal of Pain and Symptom Management [43]	Population study.	52,316 Canadian cancer patients between 2003 and 2006. 27,255 had received home palliative care services	To test the association between the quality of home palliative care services and quality of end-of-life palliative care indicators.	Increased quality of home palliative care services was associated with a lower proportion of men having 1 or more visit to an emergency department during the last month of life (risk ratio 0.924; 95% CI 0.867–0.985). In women, higher-quality home palliative care services were also associated with a higher proportion of patients dying at home (risk ratio 2.255; 95% CI 1.703–2.984) and spending less time in hospital (risk ratio 0.765; 95% CI 0.692–0.845).	Effective home palliative care services were associated with improved end-of-life palliative care indicators.	Lack of a needs assessment.
Iupati and Ensor; 2015; Internal Medicine Journal [44]	Retrospective, quantitative study of consecutive patient records over 6 years.	73 New Zealand-based patients with advanced chronic obstructive pulmonary disease who were referred to 2 community hospice programmes.	To examine the impact of community-based hospice programmes on hospital admission in patients with advanced chronic obstructive pulmonary disease.	Following entry into the hospice programme, a mean decrease of 2.375 hospital admissions over 1 year was observed.	Community-based hospice programmes may be linked with a reduction in hospital admissions in patients with advanced chronic obstructive pulmonary disease.	Highly selected and small sample size. Only 2 regional hospices.
Rosenwax et al.; 2015; Palliative Medicine [45]	Quantitative, retrospective, population-based cohort study.	All patients in Western Australia who died with dementia between January 2009 and December 2010 (*n* = 5261) and a comparator of patients who died from other conditions who received palliative care (*n* = 2685)	To report on the use of hospital emergency departments during the last year of life by patients who died with dementia and the effect that community-based palliative care had upon that use.	The rate of emergency department visits significantly increased if community-based palliative care was not being received, varying over the last year of life. During the initial 130 days, patients who received standard care visited the emergency department 1.4 more times often than those receiving community-based palliative care. In the last month of life, those receiving standard care in a private residence visited the emergency department 6.7 times more frequently and those receiving standard care in a care facility visited emergency department 3.1 times more frequently than those who received community-based palliative care.	Community-based palliative care of people who die with dementia is linked with a significant reduction in emergency department visits during the last year of life.	A retrospective study using data not collected for the purpose of the study.
Alonso-Babarro et al.; 2013; *Palliative Medicine* [46]	Quantitative population-based comparison.	549 cancer patients in 2 areas in the Madrid region. Only 1 area had a palliative home care team.	To explore the impact of palliative home care team in the last 2 months of life on place of death, emergency room visits, admissions and use of hospital resources.	Frequency of patients dying in hospital was significantly lower in the palliative home care team area (61% vs. 77%, *p* < 0.001), as were the number of patients using emergency services (68% vs. 79%, *p* = 0.004) and number of patients using in-patient services (66 vs. 76%, *p* = 0.012). After adjusting for other factors, patients in the palliative home care team area had a lower risk of hospital death than those in the non-palliative home care team area (adjusted odds ratio 0.4, 0.2–0.6).	Suggests that palliative home care team is associated with reduced in-patient deaths and use of hospital services in the last 2 months of life.	Focused on 2 specific urban areas, meaning unidentified factors may influence in-patient deaths.
McNamara et al.; 2013; *Journal of Palliative Medicine* [47]	Quantitative, retrospective cross-sectional study.	746 Australian deceased cancer patients between August 2005 and June 2006.	To investigate whether early entry to community-based palliative care reduces emergency department admissions during the last 90 days of life.	During the final 90 days of life, fewer patients who had early access to palliative care visited an emergency department than did patients without early access (31.3% vs. 52.0%).	Early access to community-based palliative care can reduce the frequency of patients’ emergency department visits.	The data were not originally collected to answer the research question.
Ranganathan et al.; 2013; *Journal of Palliative Medicine* [48]	Quantitative, retrospective cohort study.	391 American palliative home care patients compared with 890 palliative care patients receiving standard care at home, post-acute care programme.	To study the impact of palliative home care on 30-day hospital readmission rates.	Those receiving palliative care at home had a 30-day readmission probability of 9.1% vs. 17.2% in those who received standard care at home.	Compared to regular care at home, palliative home care may reduce 30-day hospital readmission.	The study could not adjust for treatment intensity, which may have affected readmission.
Cassel et al.; 2016; *Journal of the American Geriatric Society* [49]	Quantitative, retrospective study using propensity-based matching.	368 American patients who received a home-based palliative intervention, Transitions, between 2007 and 2014 were propensity matched with 1075 control individuals. The 4 disease types were cancer, chronic obstructive pulmonary disease, heart failure and dementia.	To assess non-clinical outcomes of a home-based palliative care programme.	For all 4 diseases, the number of participants admitted to hospital and the number of hospital days were lower for the Transitions group than the control (p < 0.001). The transitions group also had a lower rate of admission during the final 30 days of life (*p* < 0.001) and hospital deaths (*p* < 0.001). The mean 30-day readmission rate was lower in the Transitions group for COPD (*p* = 0.005), heart failure (*p* < 0.001) and dementia (*p* = 0.01), but not for cancer (*p* = 0.08).	The home-based Transitions programme could reduce hospital utilisation for the 4 included disease types.	Retrospective studies have the potential for selection bias.
Tan el al; 2016; *Palliative medicine* [50]	Retrospective cohort study.	321 Singaporean cancer-diagnosed patients who had an expected prognosis of ≤1 year and were enrolled in an integrated hospice home care programme were compared with 593 patients who were referred to other home hospices.	To evaluate the impact of an integrated hospice home care programme on acute care service usage and on rate of home deaths.	Hospital deaths were significantly lower in programme participants (12.1% vs. 42.7%). The programme group had significantly lower emergency department visits and hospitalisations at 30 days, 60 days and 90 days prior to death.	Integrating acute care and home hospice care could reduce acute care service usage.	Only cancer patients were included.
Riolfi et al.; 2014; *Palliative Medicine* [51]	Retrospective cohort study.	402 patients who died in 2011 of cancer as a primary cause in Veneto region, Italy.	To assess the effectiveness of appropriate palliative home care services in reducing hospital admissions and identify predictive factors in hospitalisations of patients treated at home.	The 39.9% of patients who were enrolled in the palliative care programme were more likely to die at home (53.8% vs. 7.9%), had fewer hospital admissions (0.4 vs. 1.3 admissions) and shorter stays (4.4 vs. 19.6 days) in their last 2 months of life.	The findings indicate that palliative home care teams enable consenting patients to stay at home with reduced hospital usage compared with those not under the care of palliative home care teams.	Variables between the intervention and control groups, such as symptoms and socio-economic status, were not considered.
Lukas et al.; 2013; *Journal of Palliative* Medicine [52]	Longitudinal case series.	369 patients with advanced complex illness referred for home-based palliative consultation in the mid-Atlantic United States.	To evaluate home-based palliative care on hospital outcomes within a fee-for-service environment.	There was a significant reduction in hospital utilisation (number of hospitalisations, total hospital bed days, total costs, variable costs and probability of 30-day readmission). The only exception was no change in the probability of an emergency department visit.	These results suggest a home-based palliative medicine practice may reduce hospital utilisation for patients with advanced, complex illness.	Non-randomised. Only accessed hospital utilisation data from 1 health care network.
Goldenheim et al.; 2014; *Journal of Palliative Medicine* [53]	Retrospective cohort study.	59 patients (19 readmitted within 30 days and 40 controls not readmitted) aged ≥65 years who were newly discharged to home hospice care between February 2005 and January 2010.	To identify factors associated with 30-day readmission among older adults newly discharged to hospice.	25% of admitted patients received a palliative care consultation, compared with 47% of patients who were not readmitted.	Patients discharged to hospice and readmitted within 30 days were less likely to have palliative care consultations.	Small sample and selection bias.
Kerr et al.; 2014; *Journal of Palliative Medicine* [54]	Prospective, observational database study.	149 decedents who received the home care programme. The control population (*n* = 537) was derived using propensity score matching.	To evaluate the impact on healthcare costs and utilisation of an innovative home-based palliative care programme implemented through a hospice-private payer collaboration.	Home care patients had much lower hospital admission rates, particularly in the last 6 months of life. There was no significant difference in emergency department use between home care patients and those in the control group.	Palliative care programs partnered with community hospice providers may achieve cost savings while providing care across the continuum.	Selection bias. Statistically significant differences in diagnoses and disease types between the control and intervention groups.
Pouliot et al.; 2017; *American Journal of Hospice and Palliative Care* [55]	Prospective cohort study.	123 participants with serious illness enrolled in a programme in New York.	To evaluate the effectiveness of Care Choices, a new in-home palliative care service.	The group had fewer emergency department visits (*p* < 0.001) and inpatient stays (*p* < 0.001)) after enrolment in the intervention.	An in-home palliative care programme may be a successful model that satisfies patients’ desire to remain at home and avoid hospital admissions.	Observational results and no randomisation.

^a^*The specialist palliative care teams included a core group of physicians, nurses and family physicians who provided integrated palliative care in patients’ homes*

A proportion of studies concentrated on a specific disease area, including cancer [41, 43, 46, 47, 50, 51], chronic obstructive pulmonary disease [44], dementia [45] and heart failure [38]. The others included mixed diagnoses.

All studies reported that their programme had one or more benefits over standard care, notably a significant decrease in hospital admissions [38–41, 44, 46, 48–55].

Eight of ten studies examining the impact of palliative care support in the community on emergency department attendance observed a reduction in attendance for patients enrolled in the intervention group versus standard-care patients [41–43, 45–47, 50, 55]. In one study, this effect was only seen in male patients, possibly because of greater provision of home palliative care services among those with prostate and colorectal cancer [43]. Two studies found no change in visits to the emergency department between intervention and control groups [52, 54].

Duration of hospital stay appeared to be shorter when patients were enrolled in one of the initiatives under examination [40, 43, 49, 51, 52], and fewer of those patients died in hospital [41, 43, 46, 49–51].

An editorial published in response to important findings on specialist palliative care teams’ experiences in Ontario communities, points to what could be achieved elsewhere with additional support: ‘If the associations reported by Seow and colleagues are causal, access to a palliative care team cuts hospitalisations by a third, use of emergency departments by a quarter, and risk of hospital death by a half, compared with usual care’ [41, 79].

#### 2c; out-of-hours telephone line

Access to advice via an out-of-hours telephone line was often a feature in major initiatives, such as the PEPS programme [27]. Three retrospective, quantitative studies examined hospital admission and emergency department attendance [39, 48, 56], and a fourth looked at 30-day readmission rates [46] (Table 4). All were linked to a significant decrease in hospital utilisation. Lustbader et al., found that a home-based palliative care programme including an out-of-hours telephone line was associated with a 34% reduction in hospital admission and nearly 200 fewer emergency department admissions per 1000 patients during their final month of life [39]. The benefits of an out-of-hours telephone line were also observed by Ranganathan et al. [48]. Patients enrolled in the initiative being studied had a 30-day readmission probability of 9.1%, compared with 17.2% in patients receiving standard care.

**Table 4:** 24/7 out-of-hours telephone line

Author, date and journal	Study design	Sample and setting	Research focus	Relevant results	Wider implications	Limitations
Sue Ryder; 2012; Partnership for Excellence in Palliative Support (PEPS) [27]	Longitudinal pilot study.	PEPS is a single-point-of-access, 24-h telephone service in Bedfordshire, England, which brings together 15 organisations. A senior nurse is the first point of access.	To compare information from a sample of patients with hospital activity datasets before and after registration.	1051 patients were registered. 68% who died were supported to die at home, with only 10% dying in an acute hospital.	Data suggested that introduction of a single point of access service could result in 30% fewer admissions, a 30% shorter stay and a cost reduction of around £300 per admission.	Pilot project.
Lustbader et al.; 2017; *Journal of Palliative Medicine* [39]	Quantitative, retrospective, comparative analysis.	82 US Medicare Shared Savings Program accountable care organisation deceased patients compared with 596 patients receiving usual care between October 2014 and March 2016.	To evaluate the impact of a home-based palliative care programme that included a 24/7 telephone line.	A 34% reduction in hospital admissions during the final month of life was observed in hospital-based palliative care-enrolled patients. Hospital-based palliative care was also linked with a reduction in emergency department visits per 1000 patients compared with standard care (878 vs. 1097).	Hospital-based palliative care in an accountable care organisation is linked with significant cost savings and fewer hospital admissions.	Did not test for the individual benefits or drawbacks of a 24/7 telephone line in this setting.
Alonso-Babarro et al.; 2013; *Palliative Medicine* [46]	Quantitative population-based comparison.	549 cancer patients in 2 areas in the Madrid region. Only 1 area had a palliative home care team.	To explore the impact of palliative home care team in the last 2 months of life on place of death, emergency room visits, admissions and use of hospital resources.	Frequency of patients dying in hospital was significantly lower in the palliative home care team area (61% vs. 77%, *p* < 0.001), as were the number of patients using emergency services (68% vs. 79%, *p* = 0.004) and number of patients using in-patient services (66 vs. 76%, *p* = 0.012). After adjusting for other factors, patients in the palliative home care team area had a lower risk of hospital death than those in the non-palliative home care team area (adjusted odds ratio 0.4, 0.2–0.6).	Suggests that palliative home care team is associated with reduced in-patient deaths and use of hospital services in the last 2 months of life.	Focused on 2 specific urban areas, meaning unidentified factors may influence in-patient deaths.
Ranganathan et al.; 2013; *Journal of Palliative Medicine* [48]	Quantitative, retrospective cohort study.	391 American palliative home care patients compared with 890 palliative care patients receiving standard care at home, post-acute care programme.	To study the impact of palliative home care with a 24-h telephone line on 30-day hospital readmission rates.	Those enrolled in the palliative care at home programme had a 30-day readmission probability of 9.1% vs. 17.2% in those who received standard care at home.	Palliative home care programmes with an out-of-hours telephone line may help reduce 30-day hospital admission rates.	Did not individually test the effect of the telephone line. The study could not adjust for treatment intensity, which may have affected readmission.
Purdy et al.; 2015; *BMJ Supportive & Palliative Care* [56]	Quantitative, retrospective cohort study.	3564 patients who died of end-of-life conditions in North Somerset and Somerset. 829 patients used the Delivering Choice Programme, a palliative care service with an out-of-hours telephone line, front-of- house, hospital-based, end-of-life-care discharge-in-reach nurses and end-of-life care coordination centres.	To investigate the effect of the delivering choice programme on place of death and hospital utilisation.	The phone line was associated with a lower risk of hospital admission during the last week of life only (OR 0.44, 95% CL 0.25–0.78, *p* = 0.005). There was also a risk reduction of emergency department attendance in the last week of life linked with the phone line (OR 0.34, 95% CL 0.17–0.70, *p* = 0.003).	Out-of-hours telephone lines may reduce hospital utilisation close to death.	No randomisation. Did not adjust for comorbidities. Selection bias.

#### 2d; telemedicine/teleconsultation

Two studies examining the value of telemedicine in care homes from UK and US perspectives are described in the care home initiative discussion above [36, 37]. Three additional studies attempted to analyse the influence of telehealth/telecaring on hospital utilisation among patients at the end of life (Table 5). Two were RCTs [57, 58], in which telehealth was the primary variable. The impact of both telehealth versus control, and early versus delayed use of telehealth on hospital utilisation were tested, with neither demonstrating a significant benefit. Interestingly, in Hoek et al.’s study, the mean Total Symptom Distress Score was higher in the intervention group at 12 weeks [57]. In contrast, a quantitative retrospective study where tele palliative consultations between patients and any healthcare team member were an essential part of a wider programme, witnessed a reduction in both hospital and emergency admissions [39].

**Table 5:** Telemedicine

Author, date and journal	Study design	Sample and setting	Research focus	Relevant results	Wider implications	Limitations
Hex et al.; 2018; Economic analysis of care homes: New Models of Care Vanguard [36]	Longitudinal cohort study.	141 care homes that had a telemedicine programme were compared with 25 control care homes in the north of England.	To quantify the economic benefits generated by a telemedicine programme 1 year after implementation.	Emergency department admissions decreased in the care homes using telemedicine by 4% and increased in the control homes by 7%. Of the intervention homes, nursing homes saw a 13% decrease in emergency admission and residential homes saw a 6% increase. Low intervention home usage was associated with a 17% reduction in emergency admissions, whilst high usage of homes was associated with a 10% increase.	The findings were not statistically significant and can only be taken at face value.	Limited control group. Extensive data cleaning was needed to produce useable data. The intervention was rolled out over time so there was no universal start date.
Grabowski et al.; 2014; *Health Affairs* [37]	Prospective observational study.	A chain of care homes in Massachusetts introduced telemedicine in 11 homes, covering weekday evenings and weekends.	To establish whether the lack of a physician’s presence at homes out of hours might contribute to inappropriate hospitalisations and whether switching to an on-call telemedicine physician could reduce inappropriate hospital admissions.	Both intervention and control groups saw a decline in hospitalisations, of 9.7 and 5.3%, respectively. When looking at more and less engaged homes, a significant decline in hospitalisations (11.3%) was found at more engaged care homes.	The findings suggest that nursing homes fully engaged in out-of-hours telemedicine could see a reduction in hospitalisations.	Small sample.
Lustbader et al.; 2017; *Journal of Palliative Medicine* [39]	Quantitative, retrospective, comparative analysis .	82 US Medicare Shared Savings Program accountable care organisation deceased patients compared with 596 patients receiving usual care between October 2014 and March 2016.	To evaluate the impact of a home-based palliative care programme within an accountable care organisation on cost and resource utilisation during the final 3 months of life.	A 34% reduction in hospital admissions during the final month of life was observed in hospital-based palliative care-enrolled patients. Hospital-based palliative care was also linked with a reduction in emergency department visits per 1000 patients compared with standard care (878 vs. 1097).	Hospital-based palliative care in an accountable care organisation is linked with significant cost savings and fewer hospital admissions.	Did not test for the individual benefits or drawbacks of a 24/7 telephone line in this setting.
Hoek et al.; 2017; *BMC Medicine* [57]	Two-armed, non-blinded randomised control trial.	74 Dutch home-dwelling palliative care cancer patients between May 2011 and January 2015. Patients were randomised to either an intervention group that received weekly teleconsultations or a control group that received standard care for 12 weeks.	To determine the effects of weekly teleconsultations on hospital admissions for palliative patients (secondary objective).	The was no significant difference in mean hospital admissions between the intervention and control groups (0.47 vs. 0.38, *p* = 0.60).	Suggests that weekly teleconsultations do not reduce hospital admissions for palliative patients.	Some patients eligible for the trial were not approached due to clinical considerations, which may have caused selection bias. High attrition rate.
Bakitas el; 2015; Journal of Clinical Oncology [58]	Two arm randomised study	207 patients with advanced cancer attending a National Cancer Institute Center, a Veterans Affairs Medical Centre and community outreach clinics were randomly assigned to early or late intervention.	Intervention included an in-person palliative care consultation, telehealth nurse coaching sessions and monthly follow-up. Outcomes were quality of life, symptom impact, mood, one-year survival and resource use (hospital/intensive care days, emergency room visits and death location.	Overall patient-reported outcomes were not statistically significant after enrolment or before death. Kaplan-Meier one-year survival rates were 63% in the early group and 48% in the delayed group (*p* = 0.038). Relative rates of early to delayed decedents’ resource use were similar for hospital days, intensive care days, emergency room visits and home deaths.	Early-entry participants’ patient-reported outcomes and resource use were not statistically different; however, their survival 1 year after enrolment was improved compared with those who began 3 months later	Heterogeneity and differences in unmeasured characteristics could limit practical application. Results reflect a New England population. Half of patients in the delayed group were referred for palliative care consultation ahead of time. Not all patients completed all interventions.

#### 2e; ambulance and paramedic education

Ambulance and paramedical staff are often the first healthcare professionals to respond to a palliative care emergency outside of hospital. The situations they face are frequently distressing and confusing. Many feel ill prepared [59]. There is anecdotal evidence of additional palliative care training for ambulance and paramedical staff helping them make better decisions. Unfortunately, published peer-reviewed articles are lacking. Paramedic initiatives depend not only on training, but also on adequate resources to keep someone at home following an emergency call-out, which may limit ambulance initiatives’ success.

#### 2f; integrated palliative care models

Fourteen studies examined the use of integrated palliative care and its impact on hospital utilisation. Three of those studies were RCTs (Table 6) [60–73].

**Table 6:** Integrated palliative care

Author, date and journal	Study design	Sample and setting	Research focus	Relevant results	Wider implications	Limitations
Ferrell et al.; 2015; Journal of Pain and Symptom Management [60]	Prospective, quasi-experimental.	544 American patients with Stage I–IV non-small cell lung cancer were enrolled in the study. The intervention group (*n* = 272) were reviewed at integrated care meetings. The control group received standard care (*n* = 219).	To test the effectiveness of an integrated palliative programme for cancer patients. Outcome measures included QoL, symptoms, distress and hospital utilisation.	There were no statistically significant differences in unscheduled hospital admissions between the groups in the last 2 weeks of life.	Integrated palliative may not reduce hospital admissions	The sequential design may have resulted in temporal bias. This design did not allow for establishing the exact element of the intervention that was successful.
Benthien et al.; 2018; Journal of Pain and Symptom Management [61]	Randomised controlled trial with a 1:1 ratio.	340 Danish in- and out- cancer patients were assigned to either an intervention or control group. The intervention group received standard care plus specialised palliative care enriched with a standardised psychological intervention for patients and caregivers at home, and the control group received standard care only.	To investigate whether a transition process from oncological treatment to specialist palliative care at home for patients with incurable cancer results in more patients reaching their preferred place of care and death.	The intervention group had more hospital admissions due to health deterioration (22% vs. 16%, *p* = 0.04) or an unmanageable home situation (8% vs. 4%, *p* = 0.0119). Hospital admissions were most often caused by symptoms without progression in the intervention group (11% vs. 7%, *p* = 0.0493). There was no significant difference in overall potentially avoidable admissions. Both groups felt generally safe regarding their place of care.	This type of intervention may not decrease hospital admissions in incurable cancer patients. It may be more successful for those with a slower declining rate of health and with better diagnostics at home to rule out emergencies in need of hospital admission.	Two-thirds of the control group received specialist palliative care, albeit considerably later than the intervention group. Lack of a consistent definition of potentially avoidable admissions.
Blackhall et al.; 2016; Journal of Palliative Medicine [62]	Retrospective electronic records review.	207 US patients with advanced cancer were referred to the Comprehensive Assessment with Rapid Evaluation and Treatment (CARE) Track palliative care intervention was compared with 198 deceased patients with similar diagnoses who were not.	To measure the time of referral to outpatient palliative and impact on end-of-life care for patients with cancer.	CARE Track patients had fewer end of life hospitalisations, were less likely to die in hospital and had increased hospice utilisation and decreased costs of care.	Referral to outpatient palliative care within 3 months of death improved end of life care and may reduce costs. However, many patients were not referred, and systematic methods of referrals are needed.	Non-randomised and single centre with a predominantly white population. Selection bias. Significant differences between control and intervention groups such as age and gender.
Jang et al.; 2015; Journal of the National Cancer Institute [63]	Retrospective cohort study.	5381 patients who died of advanced pancreatic cancer between January 2005 and March 2011 in Ontario, Canada. Approximately half had received a palliative care consultation.	To evaluate the impact of palliative care, including intensity, on the aggressiveness of care near death in patients with advanced pancreatic cancer.	Patients who received a palliative care consultation had lower frequencies of intensive care unit visits near death (1.1% vs. 7.8%), multiple emergency department visits (7.4% vs. 28.5%) and multiple hospitalisations (3.8% vs. 12.8%).	Palliative care is associated with less aggressive care near death.	Selection bias and lack of control of confounding variables due to observational design.
Morita et al.; 2013; Lancet Oncology [64]	Mixed-methods study.	A multifaceted programme of interventions for patients with cancer was introduced in 4 regions of Japan. Place of death was compared before (5146 decedents) and after the interventions (5546 decedents).	To assess the impact of these interventions on numbers of home deaths, coverage of specialist services and patient-reported and family-reported quality of care.	Proportions of home deaths increased significantly, from 6.76% before the intervention compared with 10.48% after intervention.	A regional programme of interventions could improve the quality of palliative care.	The data used may not be fully representative of the region.
Youens et al.; 2017; Journal of Palliative Medicine [65]	Retrospective, observational cohort study.	28,561 West Australian cancer decedents from 2001 to 2011, of which 16,530 accessed a community-based palliative care intervention.	To compare place of death and acute care hospital use in the last year of life between cancer decedents who did and did not access a community-based palliative service.	Intervention users had 3 times greater odds of dying outside of hospital than non-users. Intervention users had fewer unplanned hospital admissions and emergency department admissions in the last year/last week of life. Those who accessed the service had significantly shorter hospital stays than the control group.	The palliative care community service supported people to die outside of hospital and was associated with reduced acute care admissions, bed days and costs over the last year of life.	Limited ability to establish causation due to it being an observational study. Lack of data on symptom severity and functional status, which may have differed between groups.
May et al.; 2017; Palliative Medicine [66]	Prospective cohort study.	863 adults with advanced cancer admitted to 3 US hospitals. Usual care (*n* = 637), early palliative care (*n* = 177) and late palliative care (*n* = 49) were compared.	To establish the association between early palliative care consultation team intervention and 1) intensity of service and length of stay compared with usual care; and 2) day-to-day costs compared with a later intervention.	Early palliative care patients had shorter hospital stays than late palliative care patients (mean length of stay 6.7 vs. 13.6 days). Early palliative care patients had shorter stays than usual care patients (mean length of stay 6.7 vs. 7.8 days).	Reducing the length of stay is the biggest cost saver in early consultation for patients with advanced cancer.	Lack of control over confounding variables due to its observational design.
Rogers et al.; 2017; Journal of the American College of Cardiology [67]	Randomised controlled trial.	150 patients with advanced heart failure received standard care (*n* = 75) or standard care plus a palliative care intervention (*n* = 75) at a single centre.	To investigate whether an integrated palliative care intervention improves certain outcomes in heart failure care.	Randomisation to the intervention did not affect hospitalisation or mortality.		The single centre and intervention care was implemented by a single nurse. The control group may have received palliative care not representative of standard care.
Brannstrom et al.; 2014; European Journal of Heart Failure [68]	Randomised controlled study.	36 Swedish patients with chronic heart failure were randomised to PREFER and compared with 36 patients in a control group.	To compare the outcomes of PREFER with regard to patient symptoms, quality of life and hospitalisations with those of usual care.	The mean number of hospitalisations was significantly lower in the PREFER group (0.42 vs. 1.47). The total number of hospitalisations was lower in the PREFER group (15 vs. 53). The mean number of days spent in hospital was significantly lower in the PREFER group than in the control group (2.9 vs. 12.4).	Person-centred care combined with active heart failure and palliative care at home has the potential to improve quality of life and morbidity substantially in patients with severe chronic heart failure.	Intervention participants were significantly older than control group participants. The study was small and at a single centre.
Chan et al.; 2014; Journal of Pain and Symptom Management [69]	Prospective, longitudinal, observational study.	19 patients with end-stage renal disease who attended a renal palliative care clinic and had more than 1 emergency department visit in 3 months. Follow-up was more frequent and intensified.	To assess whether intensified and more frequent follow-up visits affected the rate of renal palliative care clinic attendance, emergency department attendance and hospital admission.	The rate of emergency department attendance (2.63 vs. 0.63) and acute hospital admission (1.59 vs. 0.58) was significantly reduced after intensified follow-up. Clinic attendance rates improved from 56 to 85%.	Intensifying renal palliative care follow-up minimised the utilisation of acute medical services and improved outpatient attendance at the renal palliative care clinic.	Small sample, selection bias. Data originally collected for a different purpose.
Horton et al.; 2013; Journal of Palliative Medicine [70]	Single-centre cohort, longitudinal, observational study	30 patients with advanced chronic obstructive pulmonary disease and 18 caregivers were followed in their home for 6 months whilst participating in an education programme followed by home-based palliative care.	To determine the feasibility of 1) implementing a customised home-based palliative care service for patients and caregivers living with advanced chronic obstructive pulmonary disease; and 2) measuring outcomes of providing such services.	25 patients and 14 caregivers enrolled in the home-based palliative care after completing the education programme. 12/17 patients who chose home as their preferred place of death. None of the deaths occurred at home. A drop in hospital utilisation was observed in the first 100 patients before vs. after enrolment.	Providing home-based palliative care for patients with advanced chronic obstructive pulmonary disease is feasible but requires further study to fulfil their place-of-death preference.	Lack of standardisation of the intervention across participants and selection bias.
Hussain et al.; 2013; International Journal of Palliative Nursing [71]	Retrospective study.	Participation in a nurse-led palliative neurology service in the North of England was offered to 62 patients. Outcomes were compared with standard National End of Life Care Programme care	To assess the key outcomes of a UK nurse-led palliative neurology service against standard National End of Life Care Programme standards.	Mean hospital admissions in the intervention group were 0.9 vs. 3.5 nationally across all diagnoses. 26% of patients receiving the intervention died in hospital vs. 46% nationally.	The service model fulfilled key standard National End of Life Care Programme recommendations and resulted in low hospital admissions and deaths.	Small sample. No control group.
Wu et al.; 2013; Journal of Palliative Medicine [72]	Retrospective cohort study.	50 US patients who received a palliative care consultation in the emergency department before hospital admission were compared with 1385 patients who received a palliative care consultation after admission to hospital.	To see whether inpatient admissions after palliative care initiated in the emergency department were associated with shorter length of stay than in patients whose palliative care was initiated after hospital admission.	Length of stay was reduced by 3.6 in patients who received palliative care consultation prior to admission.	Early initiation of palliative care in the emergency department resulted in shorter inpatient stays.	Non-randomised and selection bias.
Desrosiers et al.; 2014; Journal of Pain and Symptom Management [73]	Retrospective cohort study.	56 consecutive deaths under a new service at a hospital in Cape Town, South Africa, were compared with 48 consecutive deaths immediately before implementation of the new service.	To assess whether a hospital-based palliative care service reduces admissions and increases home death rates.	The mean number of admissions for the intervention group and control groups were 1.39 vs. 1.98. The mean total number of hospital days for the intervention and control groups were 4.52 vs. 9.3. 58.9% of the intervention group died at home, compared with 18.8% of the control group.	An outpatient hospital-based service reduced admissions and improved the chance of home death, offering a feasible and cost-effective model for such settings.	Selection bias.

*Increasing skills and knowledge of palliative care, increasing availability of palliative care services, coordinating palliative services and providing appropriate information to families and the*

The components of care varied, with 12 studies concentrating on one disease type [cancer: seven [60–66], heart failure: two [67, 68], renal failure: one [69], chronic obstructive pulmonary disease: one [70], neurodegenerative disease: one [71]].

A study by Wu et al. examined the benefits of initiating palliative care consultations in emergency departments for patients suffering from a range of terminal diseases [72], while Desrosiers et al., reported on the value of a hospital-based palliative care service for patients with advanced organ failure in sub-Saharan Africa [73].

Overall, 11 studies found the initiative to be beneficial. In contrast, Ferrell et al., in a prospective quasi-experimental study, examined the value of an integrated care group for managing patients with non-small cell lung cancer (NSCLC; *n* = 272) versus standard care (*n* = 219) [60].. No significant difference in unscheduled hospital admissions in the last 2 weeks of life was observed between the intervention and control groups.

Two RCTs painted a similar picture. Rogers et al., conducted a single-centre US study involving 150 patients with advanced heart failure. Subjects were randomised to either usual care (*n* = 75) or usual care plus nurse-implemented integrated palliative care (*n* = 75). While there were benefits in the intervention group in terms of quality of life, anxiety, depression, and spiritual well-being, there was no effect on rehospitalisation or mortality [67]. In a Danish study involving 340 cancer patients, Benthien et al., looked at the value of adding specialised palliative care and psychological interventions to standard care and found that the intervention group actually experienced more admissions [61]. These findings contrast with those of a smaller, single-centre Swedish RCT on chronic heart failure (Palliative advanced home caRE and heart FailurE care study; PREFER) [68]. Patients were randomised to an intervention group (usual care plus a person-centred palliative care programme (‘PREFER’, *n* = 36), or a control group (usual care, *n* = 36). Fewer hospitalisations and bed days were encountered in the PREFER group (15 hospitalisations, 103 days), than in the control group (53 hospitalisations, 305 days).

#### 2 g; palliative care outreach in rural areas

Delivering palliative care to patients outside urban areas is a challenge. The ‘Marie Curie Delivering Choice Programme ’ consisted of several elements and was delivered in rural Somerset, England. In a quantitative retrospective cohort study [56], Purdy et al., investigated the effects of this initiative on place of death and hospital utilisation. Of 3564 patients who died of a chronic condition, 829 used the Delivering Choice Programme service. Users of the service were at least 30% less likely to die in hospital, be admitted for an emergency or attend the emergency department than those who did not use the service. Readmissions after accessing the discharge in-reach service were low, at 6%.

Evaluation of a nurse-led navigation service to provide palliative care to older patients in rural Canada was similarly beneficial [74]. Over a two-year period, 25 adult patients and 11 family members living with deteriorating chronic illness received biweekly visits. Support included symptom management, education, advance care planning, advocacy, help with financial matters and psychosocial help. Participants were able to die in their preferred location and emergency room attendance was minimal and largely unpreventable.

#### 3a; innovations supporting early hospital discharge

The potential benefits of initiating palliative care consultations in emergency departments have been referred to above, with Wu et al., demonstrating a significantly shorter patient stay compared with a control group, of 3.6 days [72]. The study by Purdy et al., also included two ‘front-of-house’ hospital-based discharge nurses whose role included identifying patients wanting a non-hospital death and facilitating their transfer back home [56]. Additionally, the benefits of an early palliative care inpatient consultation in terms of shorter hospital stays were demonstrated by May et al., in a prospective multi-site cohort study [66]. May et al., also found that there were significant cost savings (63%) linked with a shorter length of stay. Further, beneficial effects of an inexpensive quality-of-life checklist on readmission rates were investigated by means of a prospective cohort study [75]. The researchers compared outcomes among 48 intervention subjects and 48 controls admitted to hospital with heart failure in Michigan. After adjusting for descendants, there was a significant difference in 30-day readmission in favour of the intervention group (2% vs. 20%.)

There is evidence to support the value of a hospital-based palliative care programme. In a retrospective study, Hua et al., found that patients in the intervention and control group experienced the same length of stay (6 days) but patients in the intervention group were 46% more likely to be discharged to a hospice [76]. These findings are supported by those of Horton et al. [77].

#### 3b; nurse-led inpatient initiatives

It is increasingly common for nurses to lead end of life care services, of which this review identified several examples [27, 74]. St Gemma’s Hospice in Leeds, England, supported by The Health Foundation, has taken this further by introducing four nurse-led beds for patients with less complex medical needs [78]. Data from this pilot project suggests that nurses can safely deliver end of life care, and during the study period, 50 patients achieved their preferred place of death, resulting in a reduction in hospital deaths and an estimated saving of 132 bed days.

## Discussion

### Main findings

This research has identified a wide range of initiatives which may affect inappropriate or non-beneficial hospital utilisation for people nearing the end of their life. Generally, interventions led to a reduction in emergency attendance and hospital bed days. A minority of studies found no benefits in the intervention group, while others showed an increase in hospital utilisation. Most publications reported retrospective studies with data originating from one centre. Research was focused on palliative care support in the community, hospice at home services, integrated care provision, out-of-hours telephone advice, care home education and telemedicine. Nurses and hospices were central to many innovations. To our knowledge, this is the first attempt at an overview.

### What this study adds

Consistent evidence was found of reduced hospital utilisation, after palliative care was introduced to the community programme, with several population-based studies benefitting from larger sample sizes (6000 to > 50,000 patients) [40–43]. This topic has been the subject of earlier reviews [80, 81]. In contrast, other interventions’ findings were mixed. Whereas improved nursing home education was found to be beneficial in four studies, it was not supported by a large RCT examining the INTERACT programme in the United States [32]. There was some support for telehealth, but again, this was not confirmed to reduce utilisation in two RCTs [57, 58]. We found little published evidence supporting initiatives such as palliative care training for ambulance staff or SPA services. One leading palliative care centre in the United Kingdom, known to the authors, is convinced as to the value of its SPA but there has been no robust evidence confirming a potential benefit published so far.

Two ‘negative’ RCTs require additional comment. In Hoek et al.’s study, the mean Total Symptom Distress Score was greater in the intervention group after the trial period [57]. Contributory factors may include the study’s small number of participants, the teleconsultation group’s higher baseline score, and a ‘nocebo’ effect, where symptoms become the consultation’s prime focus. Also, in their study, Benthien et al. [61], found that the intervention group had a higher admission rate. The researchers suggested that carers’ closer observation led to more symptoms being identified, and the publicly financed Danish healthcare system—where reimbursement is based on performance—may encourage hospital utilisation.

Relatively few publications examined initiatives designed to facilitate patient discharge from hospital once they had been admitted. The little evidence of these initiatives’ effects suggests that early specialist palliative care provided by doctors and nurses can reduce hospital bed days [56, 66, 72, 75]. There may even be benefits to starting such conversations in the emergency department [72]. Experience of healthcare staff, suggests that there are often multiple administrative issues delaying patients’ return home, so simple initiatives such as using an accompanying ‘red bag’ with patients’ details and medication may be helpful [34]. Only one study found that hospital-based palliative care increased the chance of discharge to a hospice [76].

As our research progressed, it seemed beneficial to investigate the link between outcomes and critical factors for introduction and sustainability, and how initiatives might be successfully upscaled or adopted elsewhere. This investigation has not been possible with the current data. The publications we examined, generally describe initiatives and outcomes but do not provide extensive detail. We suspect that contributory factors may include local need, leaders’ and team members’ enthusiasm and supportiveness, and funding.

### Study strengths, weaknesses and limitations

Our research provides a broad overview of current activity outside of hospitals and hospices, which may reduce unnecessary and inappropriate hospital attendance and admissions, and bed days. Such evidence could be of benefit for those seeking to develop new services and obtain sources of funding. The robustness of evidence examined in this review was, however, limited. Also, studies generally failed to identify whether reducing admissions was in accordance with the patient or their families wishes i.e. not all days spent in a hospital bed were ‘bad, unnecessary or unwanted.

Undertaking palliative care research is difficult, despite many patients’ willingness to participate [82]. It is therefore unsurprising that we only identified six relevant RCTs. Most of the data was collected retrospectively, risking recall bias. Also, many concentrated on one disease area, notably cancer, which may impact results’ generalisability. Some studies reported on only a few subjects and appeared to be driven by one or more enthusiastic healthcare professionals. This is evident in a paper by Pesut and colleagues who described the experiences of 25 older adults and their families in rural Canada, receiving bi-weekly home visits from an experienced nurse navigator over a 2-year period [74]. It is likely that without the involvement of similarly motivated individuals, such positive outcomes may not be reproduceable elsewhere.

Trying to unpick the contribution of each component of a complex intervention and establishing cause and effect can be problematic. These complexities were illustrated in the Marie Curie Delivering Choice Programme in rural Somerset, England, which comprised a telephone advice line, a nurse-led hospital-based discharge service, 22 coordination centres delivering multiagency care, and an electronic register to record advanced care wishes [56]. Using service evaluation to identify the effect of each component in reducing hospital bed days was difficult. Similar challenges have been encountered across most other studies.

The present study has several research limitations. We did not appraise the methodological quality of the selected studies. Further, a degree of judgement of what to include was required. For example, we did not explore general initiatives seeking to educate and advance the concept of palliative care. Such efforts were considered unlikely to have a measurable effect on hospital utilisation. Because of the search terms used and initiatives’ heterogeneous nature, it is possible that we may have missed important developments, especially those referred to in non-English publications. Our experience is that relevant data is sometimes buried in less accessible reports, and those studies often lack methodological detail. Also, whereas the five-year search period was selected to make this project manageable and was based on the assumption that earlier promising initiatives would be carried over into newer publications, the timeframe was relatively short. Finally, our research does not address quality of care outside hospital, or whether reducing bed days actually saves health systems money or simply transfers costs elsewhere. All of these issues may have influenced our conclusions.

## Conclusions

There is much innovation happening to improve end of life care. Evidence as to whether it reduces inappropriate or non-beneficial hospital utilisation is limited, sometimes contradictory and of variable quality. Many people working in palliative care are convinced that what they are doing can benefit patients and families through a reduction in unnecessary and burdensome end-of-life hospital admissions but, do not have robust evidence to prove it. Where success is claimed, we still know little about why an initiative worked and how it might be successfully transposed elsewhere. It is worth restating that not all bed days are ‘bad’ or unwanted by patients and families.

As so often in palliative care, further research is required. A notable example is the area of ambulance and paramedic education. It is hoped that the ongoing quantitative and qualitative HOLISTIC study, which looks at patient journeys toward the end of life by mapping Hospital Episode Statistics data against stakeholder interviews, may provide further insights.

## Data Availability

This manuscript relies on the documents listed under References. All other material is available upon request from the corresponding author.

## Abbreviations

HOLISTIC: HOspice-Led Innovations Study To Improve Care project
INTERACT: Interventions to Reduce Acute Care Transfers
NHS: National Health Service
NSCLC: Non-Small Cell Lung Cancer
PEPS: Partnership for Excellence in Palliative Support
PREFER: Palliative advanced home caRE and heart FailurE caRe
RCT: Randomised Control Trial
SPA: Single Point of Access
